# Hyaluronic acid versus botulinum a toxin injection in the treatment of premature ejaculation: A comparative study

**DOI:** 10.1038/s41598-025-02466-1

**Published:** 2025-05-21

**Authors:** Mohamed S. Hasan, AbdRaouf M. Almohsen, Ibrahim Mearaj, Maged T. Abd Elsalam, Mohamed L. Elsaie

**Affiliations:** 1 Department of Dermatology, Venereology and Andrology, Faculty of Medicine, Al-Azhar University, Cairo, Egypt; 2 Department of Dermatology, Venereology and Andrology, El Warrak Central Hospital, Cairo, Egypt; 3 Department of Dermatology, Medical Research and Clinical Studies Institute, National Research Centre, Giza, Egypt

**Keywords:** Premature ejaculation, Botulinum toxin, Hyaluronic acid, Sex, Health care, Medical research

## Abstract

The aim of the study was to assess safety and efficacy of botulinum-A toxin (BTX) injection into the bulbospongiosus muscle versus hyaluronic acid (HA) gel injection in glans penis for treatment of premature ejaculation (PE). The patients were randomly divided into 2 groups. Group a (n = 30) were injected with botulinum toxin type a injection in bulbospongiosus (BS) muscle at the perineum (25 units on each side). Group b (n = 30) were injected with 2 ml hyaluronic acid along the corona (proximal part) of the glans penis and frenulum. The mean IELT significantly increased from 1.78 min to 3.87 min after the BTX injection while the mean IELT was significantly increased to 7.3 min from 1.23 min before injection among the hyaluronic acid injected group (p < 0.001). The improvement of IELT was more significantly noted in the HA group than BTX group by the end of treatment. Both modalities provided a well- tolerated potential treatment in the management of PE with, HA demonstrating a higher efficacy than BTX. More high-quality, randomized prospective studies and the standardization of the inclusion criteria and the outcome assessment methods are needed in order to confirm these findings.

## Introduction

Premature ejaculation is defined as a deviation from the normal length of intravaginal ejaculatory latency time (IELT), which is the time taken from vaginal penetration to ejaculation. Control over the moment of ejaculation and sexual satisfaction of the man and woman are possible components. Premature ejaculation was classified as lifelong (primary) or acquired (secondary). Lifelong PE is characterized by its onset from the first sexual experience and remains a problem throughout life. Ejaculation occurs too quickly, either before vaginal penetration or < 1–2 min afterward. Acquired PE is characterized by its gradual or sudden onset, with ejaculation beingnormal before the onset of the problem. Time to ejaculation is short but not usually as fast as in lifelong PE^1^.

Treatment for PE has included oral medication, such as selective serotonin reuptake inhibitors (SSRIs), topical agents, and behavioral and cognitive therapy. Recently, dapoxetine, a novel fast-acting SSRI, was approved for the on-demand treatment of PE in several countries. However, SSRIs have possible SSRI-related adverse effects. Although the safety and efficacy of some treatments for PE have been reported, safer and more effective treatment options are still required^2^.

Botulinum-A toxin is a selective blocker of acetylcholine release from nerve endings and accordingly blocks neural transmission when injected into muscle. Toxin has been used to inhibit the rhythmic contraction of the BS muscle and hence was proposed as a potential treatment for PE^2^. Injectable implants containing hyaluronic acid had been successfully injected into the dermis of the glans penis just above the nerve terminal reducing tactile stimuli sensitization of receptors and could provide a modality for prolonging IELT. The ideal filling substance for soft-tissue augmentation should be biocompatible, nonantigenic, nonpyrogenic, noninflammatory, non-toxic, easy to use, stable after injection, nonmigratory, long-lasting but resorbable, natural-looking, and not too expensive^3^.

The aim of the study was to assess safety and efficacy of botulinum-A toxin (BTX) injection into the bulbospongiosus muscle versus hyaluronic acid (HA) gel injection in glans penis for treatment of premature ejaculation (PE).

## Methods

This study was conducted on 60 patients with premature ejaculation. The patients were recruited from andrology outpatient Clinics in university hospital from January 2023 to May 2024. An informed written consent was taken from all patients before enrollment in the study after approval of the Medical Research Ethics Committee.

Premature Ejaculation (PE) was diagnosed based on the Diagnostic and Statistical Manual of Mental Disorders, fourth edition DSM-IV-TR criteria, and only patients with lifelong PE were included in the study. Participants in the study were required to be in stable, heterosexual, monogamous relationships lasting more than 3 months and to be 25 years of age or older.

Patients who received any medication for premature ejaculation during the last two months, Patients on sex hormone supplementation, Erectile dysfunction, or penile prosthesis, Patients with chronic debilitating diseases e.g.: liver cellfailure, renal failure, or severe uncontrolled diabetes, Patients with a history of pelvic or spinal surgical Operations, or Patients on chemotherapy or radiotherapy or history hyper-sensitivity to any of hyaluronic acid preparationswere excluded from the study.

The 60 patients were divided into 2 groups. A sample size calculation was performed using data from a similar trial and assuming a two-tailed alpha of 0.05, 80% power, a pooled standard deviation of approximately 1.1, and a mean difference of 1.5 min between the groups, the minimum required sample size was calculated to be 9 participants per group. After adjusting for a potential dropout rate of 15%, the final recommended sample size was 11 participants per group, totaling 22 participants which we exceeded to 30 per group^4^. Independent randomization (in 1:1 ratio) was conducted by a third party (not involved in the study) using a computer-generated random table according to botulinum-A (BTX) or hyaluronic acid (HA) glans injection.

**Group A (botox injection)**: 30 male patients with premature ejaculation were injected with botulinum toxin type A injection (Allergan, Ona botulinum toxin-A IU™) in bulbospongiosus muscle at the perineum (25 units on each side). Patients were positioned in lithotomy position, the perineal area was disinfected with 70% alcohol, and the bulbospongiosus muscle was identified using ultrasound (6–13 Hz superficial probe with MAX depth 6 cm).

**Group B (hyaluronic acid injection)**: 30 male patients with premature ejaculation were injected with 2 ml hyaluronic acid (Restylane volume® IPN Like TECHNOLOGY, VIVACY Laboratories, Paris, France™) along the corona (proximal part) of the glans penis and frenulum.

All study participants were subjected to demographic data such as age, course, duration of the disease, medical history, and any history of sensitivity to the drugs as well as male dominance and sexual intimacy. All patients in this study were evaluated according to IELT in seconds by stopwatch by patients themselves pre- and post-treatment (after 2 months of injection) to determine the exact effect of both botulinum toxin and hyaluronic acid injections in this study.

All patients were evaluated pre and after-treatment according to the premature ejaculation profile (PEP) questionnaire to determine the exact effect of both botulinum toxin (BTX) and hyaluronic acid (HA) injections in this study. The PEP is a 4-question PRO that asks a respondent about his subjective sense of control over ejaculation, distress related to PE, interpersonal difficulty and satisfaction with sexual intercourse. Each question is answered on a 5-point Likert-type scale and an index score is derived by averaging the responses to the 4 questions^5^.

Evaluation and follow-up of all patients was carried out at baseline and after two months of treatment. Regarding the IELT, the partner was requested to run a calibrated stopwatch on vaginal penetration and stop it on extra or intravaginal ejaculation or withdrawal of the penis without ejaculation at the end of sexual intercourse. The IELT considered for statistical analysis was the geometric mean of the last 3 sexual acts’ IELT at each time point i.e., pre-injection and 2 months after injection. It is to be mentioned that all patients received training on how to record IELT using calibrated stopwatch prior to enrollment in the study. Sexual satisfaction of both, the patient and his wife based on the PEP questionnaire.

## Statistical analysis

Data were analyzed using Statistical Program for Social Science (SPSS) version 15.0. Quantitative data were expressed as mean ± standard deviation (SD), median, and IQR, which is the measure of statistical dispersion, being equal to the difference between the 75^th^ and 25^th^ percentile. Paired t-test of significance was used when comparing between two means. Chi-square tests: was used when comparing non-parametric data. Probability (P-value), P-value < 0.05 was considered significant, P-value < 0.001 was considered highly significant, and P-value > 0.05 was considered insignificant.

## Results

Sixty patients were involved in the study which was divided into two equal groups, group A (botulinum toxin type A injection) and group B (hyaluronic acid injection) groups. The mean age of patients was 47.48 years, with the mean duration of marriage being 17.35 years. The mean smoking index among patients was 9.17 cigarettes per day; with the mean duration of smoking was 9.8 years. The majority of patients had no history of DM (81.7%), HTN (73.3%), history of surgery (95%), or no medication use (45%). The mean intercourse frequency per week was 1.77. Sufficient intimacy was reported in 86.9% of cases (86.7% in A and 90% in B). The demographic and baseline characteristics were similar for patients in both groups. Moreover; the baselines of IELT and scores in each PEP items didn’t demonstrate no significant difference between patients in BTX and HA groups. Table 1

**Table 1:** Patient characteristics and medical history data.

Variable		Total	A (BTX)	B (HA)	p Value
No		60	30	30	–
Age in years (mean ± SD)		47.48 ± 6.68	48.83 ± 8.57	46.13 ± 3.67	0.148
Duaration of marriage (mean ± SD)		17.35 ± 8.87	19.87 ± 11.09	14.83 ± 4.90	0.043
Smoking history	Index (mean ± SD)	9.17 ± 9.07	7.00 ± 8.77	11.33 ± 9.00	0.045
	Duration in years (mean ± SD)	9.80 ± 10.48	6.67 ± 9.44	12.93 ± 10.68	0.007
Intercourse per week (mean + SD)		1.77 + 0.95	1.70 + 0.95	1.83 + 0.95	0.555
Medications (%)	No	66.7	46.7	86.7	0.658
	Insulin	5.0	10	0	
	Oral hypoglycaemic	5.0	10	0	
	Antihypertensive	23.3	33.3	13.3	
DM (%)		18.3	36.7	0	< 0.001
HTN (%)		26.7	40.0	13.3	0.033
Surgery (%)		5.0	10.0	0	0.083
Male dominance (%)		77	76.7	80	0.666
Sufficient intimacy (%)		86.9	86.7	90	0.004

DM: Diabetes mellitus; HTN: hypertension; A (BTX): Group A botulinum toxin; B (HA): Group B; hyaluronic acid; *p-value shows statistically significant difference when < 0.05; Male dominance" refers to the patient’s self-reported role as the primary initiator or decision-maker in sexual activity.

The mean IELT significantly increased from 1.78 min to 3.87 min after the BTX injection while the mean IELT was significantly increased to 7.3 min from 1.23 min before injection among the hyaluronic acid injected group (p < 0.001). The improvement of IELT was more significantly noted in the HA group than BTX group by the end of treatment Tables (2, 3, 4, 5).

**Table 2:** Comparison between IELT before and after BTX and HA injection.

*IELT in min* (mean ± SD)	Before	After	p Value
BTX group	1.78 ± 0.63	3.87 ± 0.97	< 0.001
HA group	1.23 ± 0.49	7.30 ± 0.84	< 0.001
p2 Value	0.094	< 0.001	

*p1-value shows statistically significant difference of each group before and after injection < 0.05;; BTX : botox; HA: hyaluronic acid; IELT: intravaginal ejaculatory latency time ; SD: standard deviation; p2: significance between both groups before and after injection.

**Table 3:** Average (*SD*) scores of PEP and each items of PEP in the botox group (A) before and after injection.

PEP measurement	Before	After	p Value
Ejaculation control	2.37 ± 0.72	5.63 ± 0.89	< 0.001
Distress	1.83 ± 1.08	1.73 ± 0.87	0.639
Satisfaction	2.00 ± 0.82	5.47 ± 0.57	< 0.001
Relationship difficulties	1.33 ± 1.12	1.60 ± 0.97	0.301
Total	7.53 ± 3.51	14.43 ± 3.00	< 0.001

PEP: premature ejaculation profile; SD: standard deviation; *p-value shows statistically significant difference when < 0.05.

**Table 4:** Average (*SD*) scores of PEP and each items of PEP in the hyaluronic acid group (B) before and after injection.

PEP measurement	Before	After	p Value
Ejaculation control	2.27 ± 0.74	7.43 ± 0.50	< 0.001
Distress	1.83 ± 1.08	3.50 ± 0.51	< 0.001
Satisfaction	2.00 ± 0.82	7.27 ± 0.45	< 0.001
Relationship difficulties	1.33 ± 1.12	3.43 ± 0.50	< 0.001
Total	7.43 ± 3.48	21.63 ± 0.96	< 0.001

PEP: premature ejaculation profile; SD: standard deviation; *p-value shows statistically significant difference when < 0.05.

**Table 5:** Average (*SD*) scores of PEP and each items of PEP in both groups before injection.

PEP measurement	Total	BTX	HA	p Value
Ejaculation control	2.32 ± 0.73	2.37 ± 0.72	2.27 ± 0.74	0.184
Distress	1.83 ± 1.08	1.83 ± 1.08	1.83 ± 1.08	0.999
Satisfaction	2.00 ± 0.82	2.00 ± 0.82	2.00 ± 0.82	0.999
Relationship difficulties	1.33 ± 1.12	1.33 ± 1.12	1.33 ± 1.12	0.999
Total	7.48 ± 3.47	7.53 ± 3.51	7.43 ± 3.48	0.184

*p-value shows statistically significant difference when < 0.05 ; PEP: premature ejaculation profile; BTX : botox group ; HA: hyaluronic acid group.

To assess perceived control over ejaculation, satisfaction with sexual intercourse, personal distress and interpersonal difficulty related to ejaculation of the patients and their partners, PEP was surveyed before and treatment. The mean score of ejaculation control was 2.32, with a mean of 2.37 among the BTX group and 2.27 among the HA group. The mean score of distress was 1.83 in both groups. The mean score of satisfaction was 2 in both groups. The mean score of relationship difficulties was 1.33 in both groups. All PPE scores were insignificantly different among both groups before injections Tables (4, 5).

To assess the potential confounding impact of comorbidities (DM and HTN), we performed a post-hoc sensitivity analysis excluding patients with DM and HTN. The results remained consistent, indicating that the observed differences between groups were likely attributable to treatment effects rather than baseline health disparities. Supplementary table 1.

Following treatment; the patients reported significant improvements in perceived control over ejaculation and satisfaction with sexual intercourse at the endpoint of the study compared with baseline in both groups (*p* < 0.0001) for both. The mean total PEP score was 7.53 before and 14.43 after treatment in BTX group, and 7.43 before and 21.63 after treatments in HA. There was statistically significant improvement only regarding ejaculation control and satisfaction in BTX group. However, there was evident statistically significant improvement in ejaculation control, distress, satisfaction, and relationship difficulties in HA group. Table 6

**Table 6:** Average (*SD*) scores of PEP and each items of PEP in both groups after injection.

PEP measurement	Total	BTX	HA	p Value
Ejaculation control	6.53 ± 1.16	5.63 ± 0.89	7.43 ± 0.50	< 0.001
Distress	2.62 ± 1.14	1.73 ± 0.87	3.50 ± 0.51	< 0.001
Satisfaction	6.37 ± 1.04	5.47 ± 0.57	7.27 ± 0.45	< 0.001
Relationship difficulties	2.52 ± 1.20	1.60 ± 0.97	3.43 ± 0.50	< 0.001
Total	18.03 ± 4.25	14.43 ± 3.00	21.63 ± 0.96	< 0.001

*p-value shows statistically significant difference when < 0.05 ; PEP: premature ejaculation profile; BTX : botox group ; HA: hyaluronic acid group.

The mean improvement in IELT in BTX group was 2.08 min; while it was 6.07 min in HA group. This difference was statistically significant and regression analysis showed that the improvement in IELT was dependent only on injection type. Post-micturition dribbling was experienced by 3 subjects in the BTX group and started on the 5^th^ day and was relieved by 2 months while urethral bleeding was reported in 2 subjects in the BTX group and 1 patient in the HA group and stopped spontaneously in 3 days. Table 7

**Table 7:** Logistic regression analysis of post-injection IELT compared to other factors.

Factor	Regression coefficient	SER	T Value	p Value
Age	-0.106	0.028	-1.027	0.309
Duration of marriage	-0.054	0.022	-0.487	0.628
Smoking index	-0.038	0.026	-0.285	0.777
Smoking history duration	0.159	0.024	1.131	0.263
Injection Type	0.781	0.271	10.221	< 0.001

SER: Standard error of regression*p-value shows statistically significant difference when < 0.05; Injection type refers to the intervention group: Group A (Botulinum toxin A) and Group B (Hyaluronic acid).

## Discussion

In PE, ejaculations occur very shortly of vaginal penetration. Multiple factors induce penile sensitivity including dorsal nerve distribution and density of receptors as well as hypersensitization to tactile stimuli can all affect the threshold of premature ejaculation. When erection happens, it stretches the skin and exposes more of the nerve endings embedded within the connective tissue layer covering the glans. Thus; a dense hyaluronic acid (HA) agent injected above such terminals are capable of inhibiting the hypersensitivity of such receptors providing a potential to increase ejaculatory latency time^6^.

In the current study the IELT time has significantly increased from. 1.23 ± 0.49 to 7.30 ± 0.84 min after injection with a mean IELT prolongation of 6.07 ± 0.88 min (p < 0.001). Results were comparable with a number of other studies in literature. One study compared dorsal neurectomy (group 1) versus dorsal neurectomy + HA glans injection (group 2) versus glans augmentation with HA only (group 3) in 139 PE patients and demonstrated and demonstrated an almost threefold IELT prolongation following HA injection^7^. Another study examined HA injections (in two different techniques; namely the fanning and multiple puncture techniques) in 60 patients with lifelong PE reported a significant 3.6-fold increase in the mean IELT in both groups respectively^8^.

Littara et al. performed a 1 ml HA injection in the glans of 110 PE patients and demonstrated a 3.3-fold increase in IELT at 6 months post-treatment^9^. Alahwany et al. compared injecting 1 ml of HA (n = 15) in PE patients versus saline injection in control group (n = 15) and demonstrated an IELT increase of 2.6 folds in the HA group compared to 1.1 folds increase in the saline group after 1 month of injection (p < 0.001)^10^. Another study comparing HA glans injection to saline injection revealed 4.5 folds significant increase compared to the baseline values and the control group^11^. Sakr et al. recently used 2 ml HA in glans penis via 30-gauge needle using a five-puncture technique and reported a significant sixfold IELT increase from baseline after 12 months of injection in 30 PE patients (p < 0.001)^12^.

Earlier speculations were published in 2010 signifying the inhibitory effects of using BTX for inhibiting the rhythmic contractions of the BS that may prolong ejaculatory latency and has been proposed as a potential management option for PE. Based on such hypothesis it was determined that BTX could inhibit and affect the expulsion phase of ejaculation without exerting any effect on the emission phase^13^.

In the current study the IELT time has significantly increased from. 1.78 ± 0.63 to 3.87 ± 0.97 min after injection with a mean IELT prolongation of 2.08 ± 0.80 min (p < 0.001). Only few animal studies reported an improvement of ejaculatory latency injected with BTX injections^14,15^. One study used BTX injections percutaneously in BS of male rats to increase their ejaculatory latency time; however, this increase wasn’t significantly remarkable when compared to control group male rats^13^. While in the other study the increase in ejaculatory latency was significantly longer when compared to control group (mean ± SD = 1092 ± 657 s, versus 298 ± 81 s, respectively)^15^.

Very few clinical trials assessed BS injection of BTX as a management potential in PE^16,17^. In 2018; Li et al. demonstrated a significantly longer IELT at 4 weeks post treatment with 100 units of BTX in 70 lifelong PE patients when compared to a control group injected with saline. The study also had significant improvement in PEP-ejaculation scores, PEP-sexual satisfaction, PEP-PE-related distress, and PEP-PE-induced difficult relationship with the partners^16^.

A recent study assessed injection of 100 units of BTX in 60 lifelong PE patients compared to a control group whom received only saline injections. The primary outcome was to compare both groups for changes in the Premature Ejaculation Profile (PEP), Intravaginal Ejaculatory Latency Time (IELT) and partner’s satisfaction at 1, 3 and 6 months after intervention. Herein, we identified a significant improvement in the PEP score in the treatment group compared to the baseline. The improvement in the PEP score was not associated with a statistically significant improvement in the IELT or female partner’s satisfaction. However, they did not identify a significant difference between the botulinum-A toxin-treated group and the placebo group in any of the tested scores^17^.

Another study demonstrated a notably extended intravaginal ejaculatory latency times in 49 lifelong PE patients who received BTX injections when compared to their initial performance after treatment. In addition, there were enhancements in PEP scores, and notably, no significant complications were reported^18^.

Adverse reactions were minimal and merely self-limiting. The main adverse events were local discomfort, ecchymosis, all of which were reported to resolve spontaneously. Three patients in the BTX group experienced transient post-micturition dribbling, which resolved within two months. This may be associated with temporary alterations in muscle tone or function following BTX injection into the bulbospongiosus muscle. Regarding urethral bleeding, (2 in BTX group and 1 in the HA group), it resolved spontaneously within three days with no further intervention.

The study is limited by being uncontrolled and relatively short follow-up of 2 months. While this duration allowed for initial assessment of treatment efficacy, it does not provide insight into long-term durability or potential late-onset adverse events.

In addition, botulinum toxin’s mechanism of action involves inhibition of acetylcholine release at neuromuscular junctions, leading to temporary muscle relaxation. The clinical effects typically last for 3–6 months depending on the injection site and dosage, after which muscle reinnervation may occur.

Patients who received any treatment for PE in the last two months were excluded, we did not stratify patients based on previous responsiveness to medical therapy. This represents a limitation in identifying whether injection therapy should be reserved as a first-line or second-line approach. Furthermore; the lack of blinding for both participants and outcome assessors introduced potential bias, especially for subjective measures such as the Premature Ejaculation Profile (PEP) questionnaire.

## Conclusion

In conclusion, while both hyaluronic acid (HA) and botulinum toxin (BTX) were effective in treating premature ejaculation, our study highlights the comparative efficacy of these treatments, with BTX showing a statistically significant improvement in IELT compared to HA. Further research is necessary to confirm these findings across larger cohorts.

## Supplementary Information

Supplementary Information.

## Data Availability

The data that support the findings of this study are available from the corresponding author upon reasonable request.

## Abbreviations

IELT: Intravaginal ejaculatory latency time
HA: Hyaluronic acid
BTX: Botulinum a toxin
PE: Premature ejaculation
SSRI: Selective serotonin reuptake inhibitor
PEP: Premature ejaculation profile
BS: Bulbospongiosus muscle
DSM-IV-TR: Diagnostic and statistical manual of mental disorders

## Code availability

All authors confirm all data and materials as well as software applications or custom code support their published claims and comply with field material.
