# Supplementary material for: Hyaluronic acid versus botulinum a toxin injection in the treatment of premature ejaculation: A comparative study
Source: Sci Rep. 2025 May 21;15:17575. doi: 10.1038/s41598-025-02466-1 (PMC12095612; doi:10.1038/s41598-025-02466-1)
Supplement: Supplementary file 1 — Supplementary Information. [file 41598_2025_2466_MOESM1_ESM.doc]

**Supplementary Table 1**: Post-Hoc Sensitivity Analysis Excluding Patients with Diabetes Mellitus and Hypertension

| **Outcome Measure** | **Botulinum Toxin A Group (n =30)** | **Hyaluronic Acid Group (30)** | **p-value** |
| --- | --- | --- | --- |
| **Baseline IELT (seconds)** | 42.3 ± 10.5 | 41.6 ± 11.2 | 0.74 |
| **IELT at 2 months (seconds)** | 106.2 ± 24.8 | 84.7 ± 20.5 | 0.001 |
| **Mean Change in IELT** | +63.9 ± 22.4 | +43.1 ± 19.2 | 0.003 |
| **Patient Satisfaction (VAS 0–10)** | 8.2 ± 1.1 | 6.7 ± 1.4 | 0.005 |

Values are presented as mean ± standard deviation. Statistical significance was assessed using independent-samples t-test. A p-value < 0.05 was considered statistically significant. **IELT**: Intravaginal Ejaculatory Latency Time; V**AS**: Visual Analog Scale
